# Toxicity Testing from the Bottom Up: Proposed Protocol for Screening Pollutants Linked to Insulin Resistance

**DOI:** 10.1289/ehp.121-A343

**Published:** 2013-12-01

**Authors:** Carol Potera

**Affiliations:** Carol Potera, based in Montana, has written for *EHP* since 1996. She also writes for *Microbe*, *Genetic Engineering News*, and the *American Journal of Nursing*.

Impaired insulin resistance (IR) is a precursor of type 2 diabetes, which is on the rise. A review of 23 studies in this issue of *EHP* investigates the effects of exposure to pollutants on IR.^1^ Based on methods described in the 23 papers, the researchers constructed a four-level protocol for IR toxicity testing, a streamlined scheme that offers a starting point for future IR–pollutant research.

The investigators modeled their plan after a conceptual framework proposed by the Organisation for Economic Co-operation and Development for evaluating endocrine-disrupting chemicals.^2^ Included among the larger family of endocrine disruptors are “metabolic disruptors,” which perturb metabolic signaling and contribute to diabetes, obesity, and other metabolic disorders.^3^

Most of the studies reviewed used a “top-down” experimental design, meaning they first looked for outcomes in whole animals and then assessed effects at lower levels of biological organization—molecules, cells, tissues, and organs. The review authors point to the need for more “bottom-up” experiments that focus on molecular events that precede IR development. Once investigators identify potential inducers of IR, they can assess higher-level effects of these pollutants.

A uniform bottom-up testing scheme could lower costs, save time, and reduce the number of laboratory animals needed to identify IR-inducing pollutants.^1^ “We need to look for molecular targets and cellular events underlying IR and use this information to build an array of assays to efficiently screen thousands of pollutants without having to sacrifice a lot of animals,” says lead author Tine Hectors, a toxicologist at the University of Antwerp, Belgium, at the time of the research (she is now at the University of California, Irvine).

Level 1 of the proposed scheme covers mechanistic information, such as gene and protein expression or insulin signaling pathway data. Level 2 includes tissue and cellular assays, such as measuring insulin-stimulated glucose uptake in cells known as primary adipocytes. More in-depth studies in key organs, such as monitoring insulin sensitivity in skeletal muscle, form Level 3. And whole-animal testing of pollutants falls into Level 4.^1^

**Figure:**
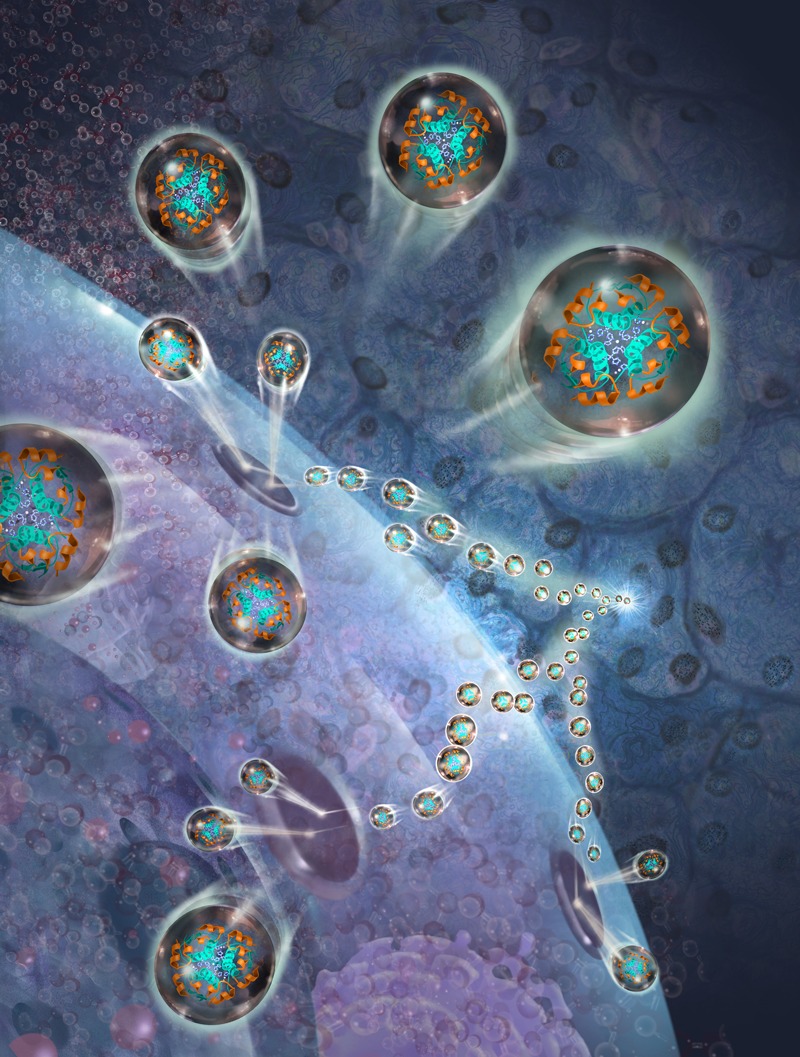
“Insulin resistance” means insulin molecules (shown here in blue and orange) are unable to enter cells, resulting in a build-up of unprocessed glucose molecules (pink) in cells. © Jim Dowdalls/Science Source

The goal of bottom-up testing is to identify potential metabolic disruptors that deserve more in-depth testing in animals, similar to screens for carcinogens and mutagens. Hectors suggests that this bottom-up screening approach could be used to identify potentially harmful metabolic disruptors that warrant further scrutiny by regulatory agencies. Analysis of molecular targets of metabolic disruptors also may reveal new drug targets to treat IR.

However, Hectors cautions that pollutant-induced IR is still a very new area of toxicology. “We are very far away from developing prevention and intervention strategies,” she says.

“Metabolic disruptors are an emerging concept that is gaining increasing attention, and Hectors’ paper offers a thorough review of the state of the science linking metabolic disruptors with insulin resistance and diabetes,” says Bruce Blumberg, a professor of developmental and cell biology at the University of California, Irvine, who was not involved in the review. Hectors and colleagues not only highlight the strengths and weaknesses of current screening assays but also suggest future *in vitro* assays that could accurately capture metabolic end points, Blumberg says.

Nearly all people with type 2 diabetes have IR.^4^ In 2010 an estimated 25.6 million U.S. residents over age 20 had diagnosed or undiagnosed diabetes, and the direct and indirect costs of diabetes in the United States reached $174 billion in 2007.^5^ Worldwide, the prevalence of diabetes doubled between 1980 and 2008.^6^

IR is involved not only in diabetes, but also obesity, metabolic syndrome, liver disease, and heart disease.^7^ A better understanding of the molecular and cellular events underlying IR could apply to these related disorders as well, or help to guide health and economic policy.
